# A Genome-Wide Gene Function Prediction Resource for *Drosophila melanogaster*

**DOI:** 10.1371/journal.pone.0012139

**Published:** 2010-08-12

**Authors:** Han Yan, Kavitha Venkatesan, John E. Beaver, Niels Klitgord, Muhammed A. Yildirim, Tong Hao, David E. Hill, Michael E. Cusick, Norbert Perrimon, Frederick P. Roth, Marc Vidal

**Affiliations:** 1 Department of Cancer Biology, Center for Cancer Systems Biology (CCSB), Dana-Farber Cancer Institute, Boston, Massachusetts, United States of America; 2 Department of Genetics, Harvard Medical School, Boston, Massachusetts, United States of America; 3 Howard Hughes Medical Institute, Boston, Massachusetts, United States of America; 4 Applied Physics Program, Division of Engineering and Applied Sciences, Graduate School of Arts and Sciences, Harvard University, Cambridge, Massachusetts, United States of America; 5 Department of Biological Chemistry and Molecular Pharmacology, Harvard Medical School, Boston, Massachusetts, United States of America; University of Toronto, Canada

## Abstract

Predicting gene functions by integrating large-scale biological data remains a challenge for systems biology. Here we present a resource for *Drosophila melanogaster* gene function predictions. We trained function-specific classifiers to optimize the influence of different biological datasets for each functional category. Our model predicted GO terms and KEGG pathway memberships for *Drosophila melanogaster* genes with high accuracy, as affirmed by cross-validation, supporting literature evidence, and large-scale RNAi screens. The resulting resource of prioritized associations between *Drosophila* genes and their potential functions offers a guide for experimental investigations.

## Introduction

A challenge in systems biology is to assign functions to genes from the information in large-scale datasets, maximizing the utility of available information to make predictions of function with verifiable performance. As initial maps of protein-protein interaction networks, gene expression profiles, and other large-scale dataset types have become available for several model organisms [1]–[15], machine-learning algorithms—including Bayesian network, Markov random field, decision tree, and Support Vector Machine (SVM) approaches—have been applied to these datasets to predict gene function [16]–[25].

Most function prediction models currently available [16]–[19], [21]–[25] in some way incorporate supervised machine learning [26]. In supervised machine learning, a series of ‘features’ describing the relationships between either two genes or a gene-function pair are calculated using known properties of the genes or corresponding proteins, such as the shortest path in protein-protein interaction networks or the correlation in gene expression profiles. A set of positive (known to exist) and negative (known/expected to not exist) gene-function pairs, along with calculated features for corresponding genes, are then designated as the training data. In the learning step, the model generalizes a classifier from the training data, and uses this classifier to predict a class label for each instance of input data provided to the model. Generally the class label is either ‘true’ or ‘false’ for a gene-function pair, although in some models an intermediate classifier is also trained to assign a label of ‘sharing function’ or ‘not sharing function’ for a pair of two genes [22], [23]. The performance of these models is usually evaluated by the “hide-and-discover” strategy of cross-validation, in which the knowledge space of gene-function pairs is randomly split into a training set and a test set. The model is trained on the data in the training set, and then used to predict functions of genes in the test set by classifying gene-function pairs with ‘true’ or ‘false’ labels. Performance is measured by comparing predictions and real gene-function associations in the test set.

It can be useful to optimize the importance of features for different functional categories. For example, while co-expression of two genes can indicate shared functions between the two genes in embryonic developmental processes, it is less informative for cytoskeleton functions, which rely more heavily on physical interactions between proteins.

To generate a resource of gene function predictions for *Drosophila melanogaster*, we applied an approach which used biological relationships to train individual classifiers for each specific functional category (here either a GO term or a KEGG pathway), thereby optimizing the importance of each feature extracted from different biological datasets for the prediction of each function. The datasets we used to calculate features were protein-protein interaction networks[27], gene expression profiles [7], [28], genetic interaction datasets [29], [30], conserved protein domains [31] and cross-species sequence similarity based on BLAST analysis [32], [33]. To train our classifier we used a Random Forest algorithm [34], which constructs an ensemble of Decision Tree classifiers. The natural resistance of Random Forest to over-fitting and its excellent performance tackling large-scale datasets with multiple features [35] makes it a good candidate for function prediction in *Drosophila*.

To evaluate prediction performance, we used a typical 10-fold cross-validation [36] to analyze the sensitivity/specificity and precision/recall characteristics of the model. We examined the reliability of our predictions against literature evidence, and compared our prediction results against positive and negative hits available in the genome-wide RNAi screening data obtained at the Harvard Medical School *Drosophila* RNAi Screening Center (DRSC, http://flyrnai.org) [37]. The considerable overlap between our prediction results, RNAi screening datasets and other literature evidence indicates that our list of prioritized *Drosophila* gene-function associations can serve as a guide for future experimental investigations, including identification of false-negatives in RNAi screens.

## Results

### GO terms and KEGG pathway membership prediction for *Drosophila* genes

The Gene Ontology (GO, www.geneontology.org) [38] and the Kyoto Encyclopedia of Genes and Genomes (KEGG) [39] were our two major sources for functional annotations. As of November 2009, Gene Ontology and FlyBase (www.flybase.org) [30] had annotated around 11,000 *Drosophila* genes with over 6,000 GO terms among the three GO branches: biological process, molecular function, and cellular component. Here we focused on the biological process (BP) branch. Each association of a gene and a GO term is labeled with some GO evidence codes [38], which denote the sources from which the association was learned. For the KEGG side, 143 pathways were assigned to 2241 *Drosophila* genes as of November 2009 (www.genome.jp/kegg). We also included three well-known pathways not yet represented in KEGG—JNK Signaling, Insulin/AKT and Hippo pathways—based on information from The Interactive Fly Database (http://www.sdbonline.org/fly/aimain/1aahome.htm) [40].

To avoid potential circularity we filtered the GO-gene associations in the training set by GO evidence codes. All GO term associations derived from non-experimental (IEA/RCA evidence codes. IEA: Inferred from Electronic Annotation; RCA: inferred from Reviewed Computational Analysis) or non-machine-traceable sources (TAS/NAS/ND/IC evidence codes. TAS: Traceable Author Statement; NAS: Non-traceable Author Statement; ND: No biological Data available; IC: Inferred by Curator), or from the datasets we used to calculate the features used in the prediction (IEP/IPI/IGI/ISS evidence codes. IEP: Inferred from Expression Pattern; IPI: Inferred from Physical Interaction; IGI: Inferred from Genetic Interaction; ISS: Inferred from Sequence or Structural Similarity) were removed, leaving only associations with IDA (inferred from direct assays) and IMP (inferred from mutant phenotypes) codes to be used as true positives. To remove GO terms that were too broadly defined (e.g., “biological process” or “cellular process”), we excluded GO terms to which 500 or more *Drosophila* genes had been assigned.

To train the prediction model we calculated features from four large-scale biological datasets: protein-protein interactions, co-expression, genetic interactions, conserved protein domains and sequence similarity. For each functional category (either a GO term or a KEGG pathway), a number of “features” were derived to describe the *a priori* similarity between the gene in question (candidate gene) and those genes known to belong to the given functional category before the prediction (reference genes). Each feature consisted of the average, maximum and minimum values of a specific measurement between the candidate gene and all reference genes in the given function category. These features were pair-wise shortest paths (the minimum number of steps needed to connect one node to the other one on a network) in the protein-protein interaction network and in the genetic interaction network, correlation of expression profiles in microarray expression datasets, correlation of genetic interaction profiles, number of shared protein domains, correlation of protein domain profiles, and sequence similarity.

### Evaluating the performance of GO term and KEGG pathway prediction model

**Figure 1:**
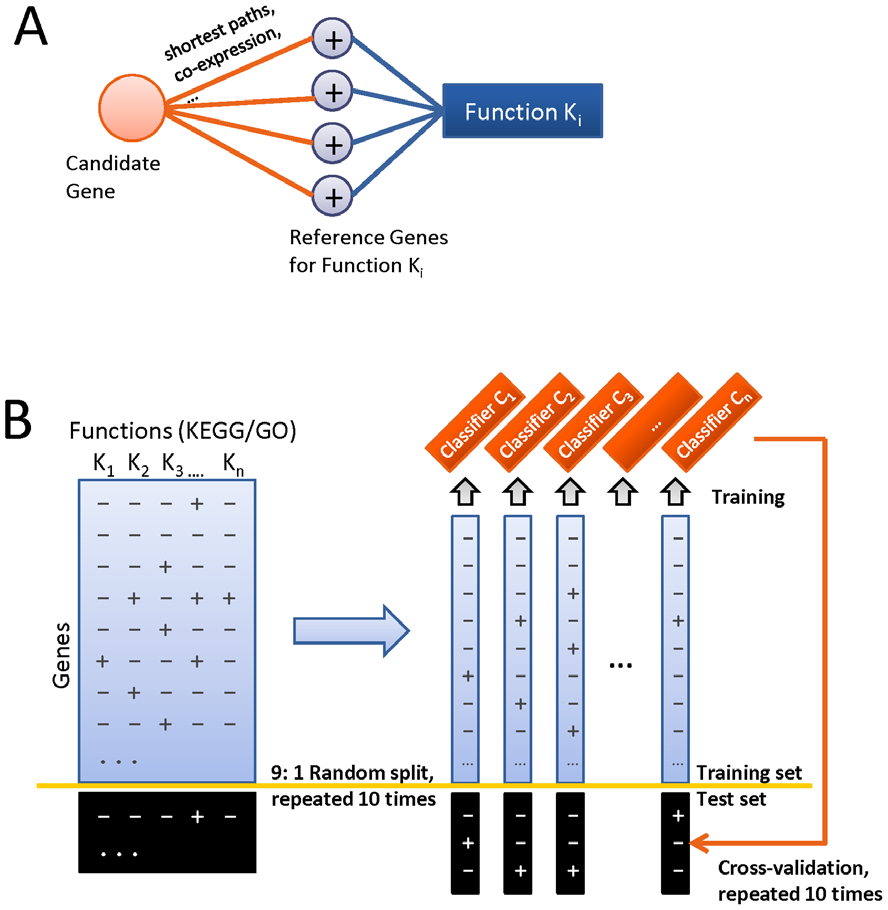
Overview of the Function-Specific Classifier model. **A**. To train the model, features describing relationships between each given candidate gene and reference genes for a given function K_i_ were derived from large-scale biological datasets. **B**. Results were evaluated by 10-fold cross-validation.

We used 10-fold cross-validation to evaluate the performance of our model. All the combinations of annotated genes and functional categories were randomly split into ten subsamples, taking one subsample as the test set and keeping the rest as the training set. A list of classifiers (one classifier for each function) were trained by the Random Forest Algorithm [34]. These trained classifiers were then used to calculate the confidence score of true association for each gene-function pair in the test set. We repeated the process ten times so that each subsample was used once as the test set (Figure 1).

**Figure 2:**
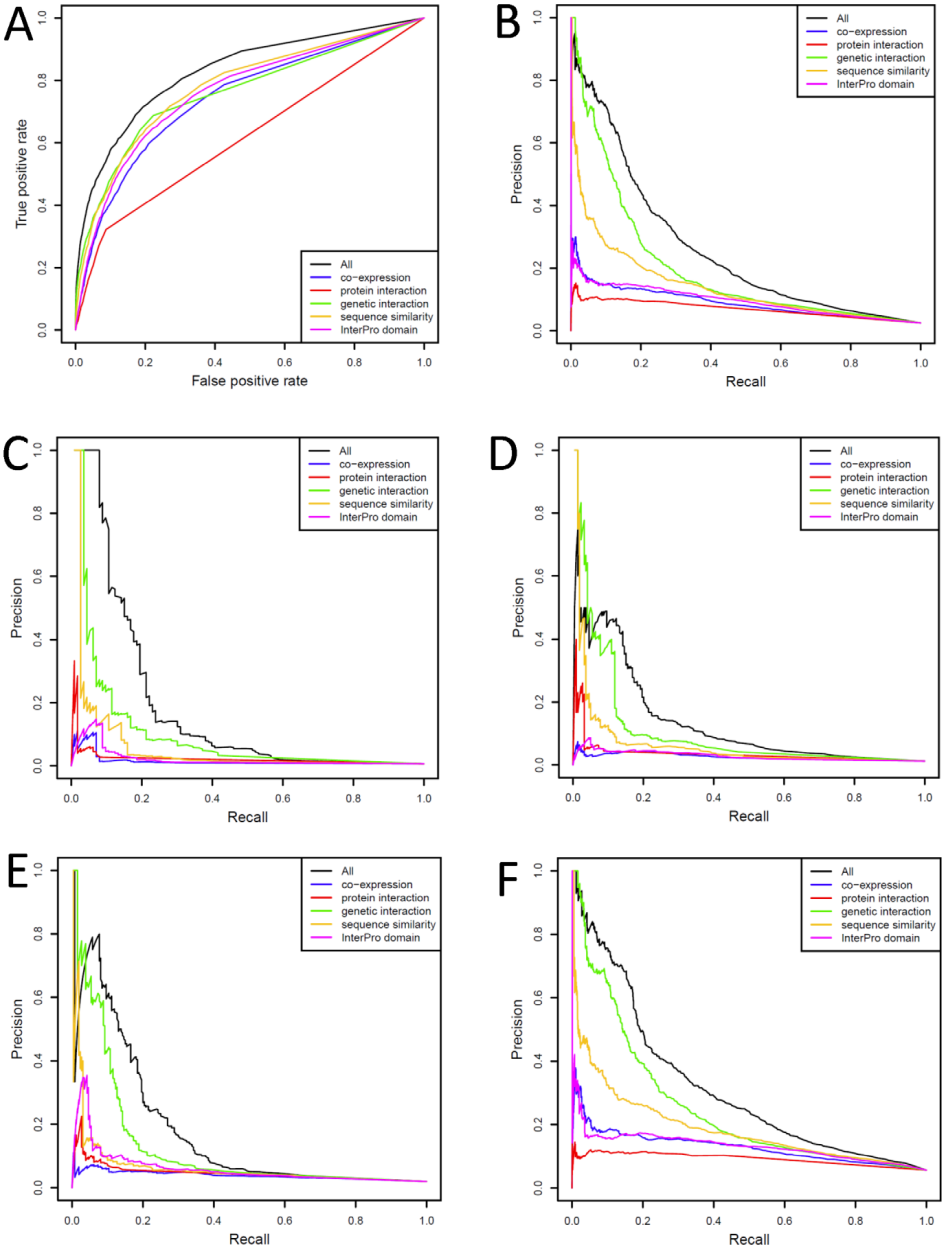
Performance of the GO term prediction model. Receiver Operating Characteristic curves (**A**) and Precision-Recall curves (**B**) for the overall performance and contribution of each feature in GO term (biological process, BP) prediction. Precision-Recall curves for the GO term prediction model for GO terms with various degrees of specificity, i.e., those that have been annotated with 2–25 genes (**C**), 25–50 genes (**D**), 50–100 genes (**E**), and 100–500 genes (**F**).

**Figure 3:**
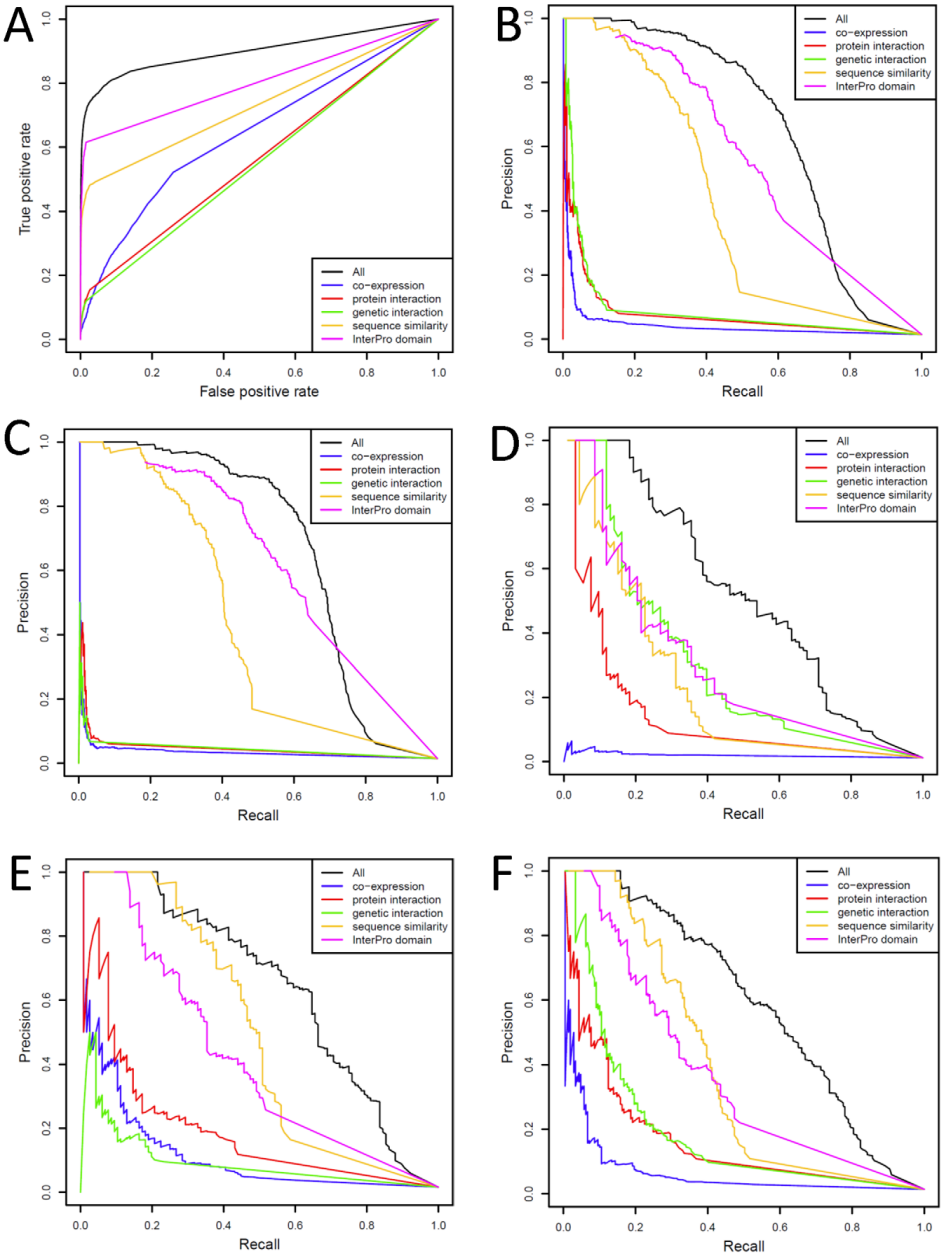
Performance of the KEGG pathway prediction model. Receiver Operating Characteristic curves (**A**) and Precision-Recall curves (**B**) for the overall performance and contribution of each feature in the KEGG pathway prediction. Precision-Recall curves for the performance of the model in predicting metabolism only (**C**), signaling pathway only (**D**), basic functions (**E**), and all non-metabolism functions (**F**).

We evaluated the performance of GO term prediction and KEGG pathway membership prediction with Receiver Operating Characteristic curves (ROC) and Precision-Recall (PRC) curves (Figures 2, 3). We examined the performance of each independent feature alone, as well as the effect on performance of leaving each feature out (Supplemental Figures S3, S4).

For GO term predictions, genetic interactions were the most informative, followed by sequence similarity, while conserved protein domains, protein-protein interactions and expression profiles contributed less (Figure 2A,B). The trend was similar when we assessed the model performance against different levels of GO term specificity defined by the number of genes associated with each GO term (Figure 2C–F). Given that all true associations in our test sets were derived from direct experiments (inferred from either mutant phenotypes or direct assays), the high performance of genetic interactions and sequence similarity features in the cross-validation indicates that many experiment-derived GO term associations could have been learned through systematic analysis of the genetic interaction network and phylogenetics of *Drosophila* genes.

For KEGG pathway membership prediction we classified all KEGG pathways into three groups: metabolism, signaling, and basic functions (Supplemental Table S1). We evaluated the performance in each group as well as the overall performance. The conserved protein domains and sequence similarity features offered the highest overall performance, despite removal of homology within KEGG Orthology (KO) groups (groups of gene orthologs sharing similar functions that have been annotated with the same conserved pathway by KEGG) from the model. That the sequence similarity feature remained highly informative suggests that the KO groups could be further expanded (Figure 3A,B), especially for metabolism and basic function categories (Figure 3C,E). For signaling pathway predictions, genetic interactions provided the best performing feature (Figure 3D), while sequence similarity scored second. We noted that the conserved protein domains could owe some performance success to the fact that genes in certain KEGG pathways may have been placed in the same pathway by virtue of sharing a common protein domain, thereby introducing some level of circularity in the performance benchmarking. This illustrated the profound difficulty of completely eliminating dependency between gold standard training examples and features used in prediction. However, even without the conserved protein domains feature, the model still performed well (Supplemental Figure S4).

The lower performance of genetic interactions in the non-signaling function categories, especially the metabolism pathways (Figure 3C), can be explained by the lower coverage of genetic interaction data for genes with these functions in *Drosophila*. Genetic interactions have been commonly used to investigate signaling pathways of *Drosophila*, as mutating many of these pathway genes produces phenotypes with significant visible effects (e.g., abnormal wings or other developmental defects). Genetic interaction analysis has been less commonly applied to study metabolism in *Drosophila*, with this lack being evident in *Drosophila* GO term annotations— among 1064 GO annotations (unpropagated, as of February 2009) with the keyword “metabolic” or “metabolism”, only two of them (*gig* and *Tsc1*, both assigned to “lipid metabolic process”) were inferred from genetic interactions (IGI tag) [30], [38]. Indeed, when we restricted the search space of our test set within genes with genetic interactions only, the performance of genetic interactions in KEGG pathways did increase considerably (Supplemental Figure S5).

In summary, genetic interactions, sequence similarity and conserved protein domains contributed substantially to prediction success, whereas expression and protein-protein interactions served to improve the performance in the high-precision region (top-left of the Precision-Recall curve). The higher performance of these features is consistent with them directly capturing function sharing between genes, as 1) genetic interactions mostly describe the phenotypes of knocked-out or knocked-down genes, 2) the sequence similarity feature transfers experimentally proven function associations from orthologs in other species to the candidate genes, and 3) many conserved protein domains have long been characterized as regions indispensible for protein functions. The lower contribution of the protein-protein interactions in our prediction can be explained by the low coverage of large-scale protein-protein interaction datasets currently available due to technical limitations and incompleteness of sampling, which has also been observed for other model organisms [15], [41]–[43]. It is possible that protein-protein interactions for *Drosophila* could become much more useful in function predictions when they have been more comprehensively sought.

### Prediction results and comparison with large-scale RNAi screening hits

We applied the trained model to make predictions within the entire unknown space of gene-to-GO term and gene-to-KEGG pathway associations, and sorted all the newly predicted gene-term associations according to the confidence of each association given by the prediction model. Predictions with confidence scores higher than 0.1 for KEGG pathway associations and higher than 0.2 for GO term associations are listed in Supplemental Table S2. All GO term association predictions are also readily accessible and downloadable via the FuncBase website (http://func.med.harvard.edu/) [44].

**Figure 4:**
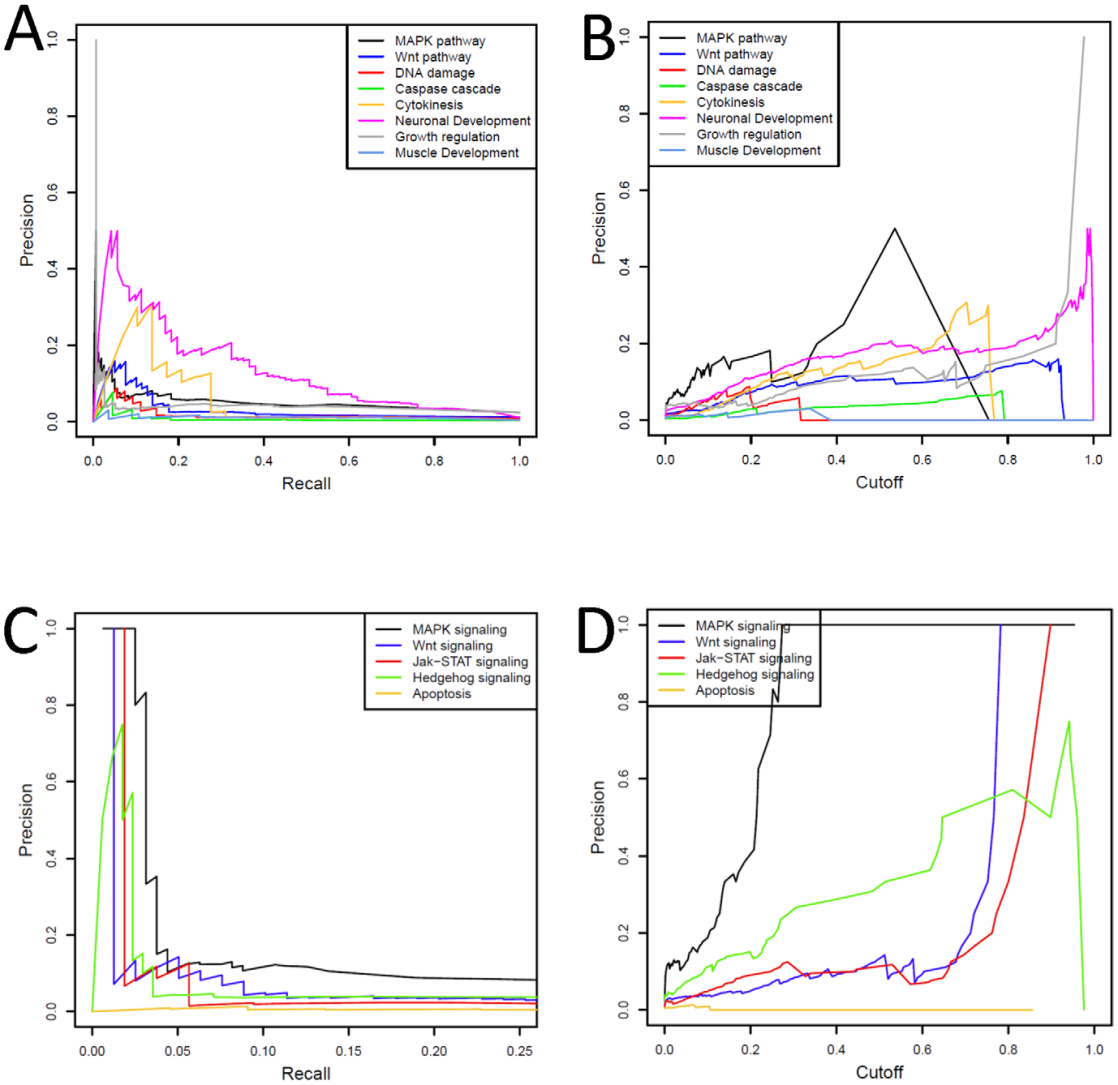
Assessing prediction quality against RNAi screening results. Precision-Recall curves (**A**) and the curves for precision vs. confidence score threshold (**B**) for the quality of GO term prediction measured by DRSC RNAi screening results; Precision-Recall curves (**C**) and the curves for precision vs. confidence score threshold (**D**) for the quality of KEGG pathway membership prediction measured by DRSC RNAi screening results.

To systematically evaluate the quality of these predictions we turned to genome-wide assays of gene function available from the *Drosophila* RNAi Screening Center at Harvard Medical School (DRSC), where RNAi screens are undertaken to systematically interrogate signaling pathways (e.g., MAPK, Wingless/Wnt, Hedgehog, JAK/STAT, and JNK pathways). These RNAi datasets are independent from the datasets we used for function predictions (protein-protein interactions, expression profiles and genetic interactions). Our trained model was applied to all gene-pathway pairs tested in these RNAi screens. We then assessed the quality of our predictions against the strong and medium signals in DRSC RNAi screening results as the positive reference set, and the non-signal results as the negative reference set, with the performance of our model assessed as precision vs. recall and precision as a function of cutoff score (Figure 4). Given the limited sensitivity inherent to RNAi experiments [45], the performance gauged by RNAi screens was, as expected, lower than that found by cross-validation. However, predictions with high confidence scores achieved high precision, indicating that our top predictions were well supported by RNAi experiments and hence likely to be biologically relevant (Figure 4B,D).

**Figure 5:**
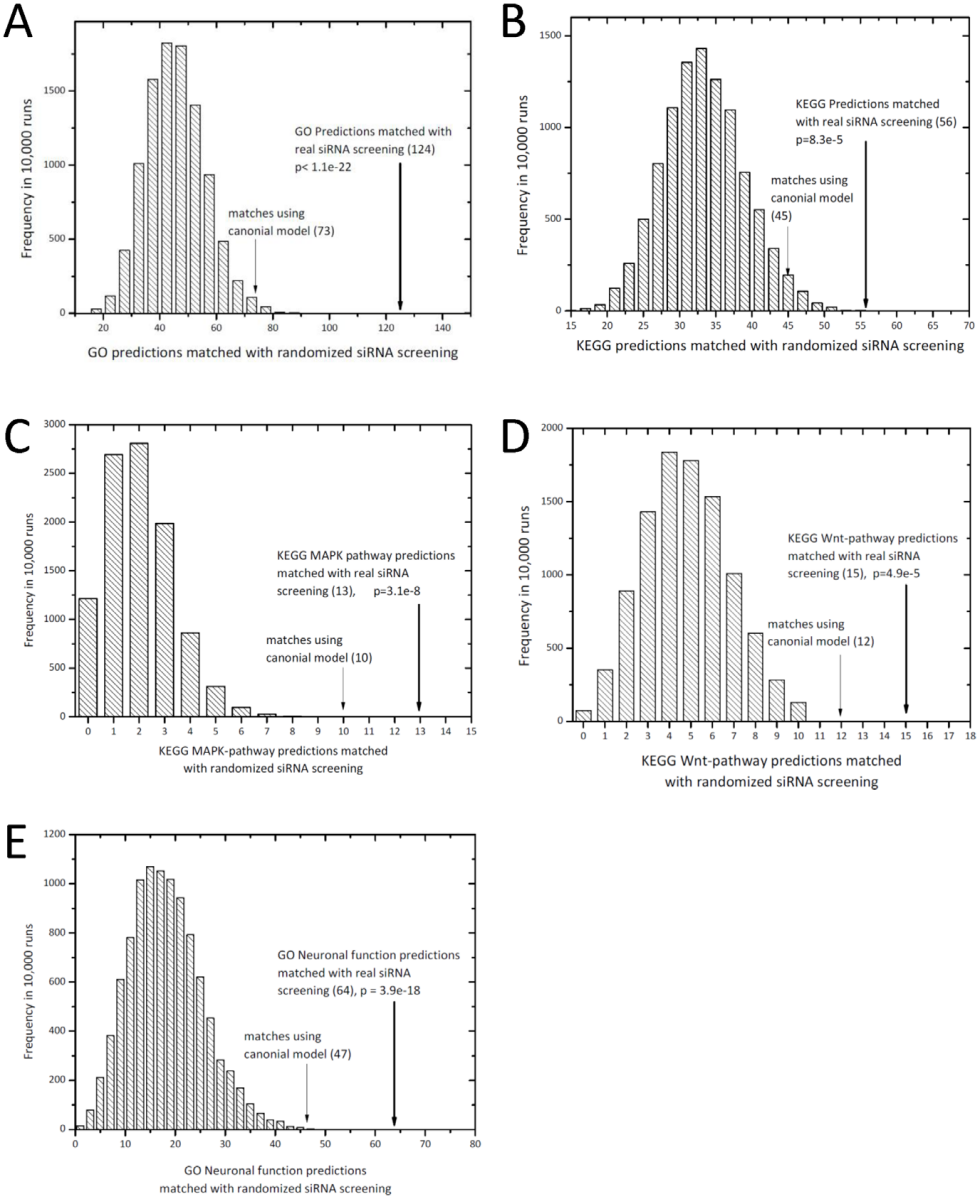
Comparison between GO term/KEGG pathway prediction and DRSC RNAi screening hits. **A–B**, GO/KEGG predictions matched with RNAi screen results compared to randomized RNAi screen data. **C–E**, individual pathway/function predictions matched with RNAi screen results compared to randomized RNAi screen data. For comparison, we show performance of a supervised machine-learning model trained using the same algorithm and datasets except that it aggregates all GO terms/KEGG pathways in its training as has been traditionally done.

To evaluate the statistical significance of the overlap between our predictions and RNAi screens, we labeled the top 0.5% of the prediction results ranked by confidence score as positive predictions, and compared them to RNAi screening data from DRSC (Supplemental Tables S3, S4). For each gene found with a strong or medium signal in RNAi screens, we examined whether we had a positive prediction for the corresponding function on a keyword-matching basis. We then compared the total number of matched associations against randomized RNAi screening data. We found significant overlap (compared to randomized RNAi data, *p*<10^−5^; Figure 5) between our computational predictions and the RNAi screening results, either in specific functions/pathways (Wnt and MAPK pathways, as well as neuronal functions) or in overall correspondence between the two studies. The performance of a canonical supervised machine-learning model, trained on the same prior features and training space we used for our function-specific classifier model and differing in that it aggregates training data for all GO terms, is shown for comparison (Figure 5). The predictions from our function-specific classifier model had higher overlap with RNAi results than the canonical model in all situations examined.

Our prediction results also provided a way to identify potential false negatives in RNAi screens. In the JAK-STAT pathway the genes *upd3* and *BRWD3* were scored 0.632 and 0.530, ranked 5th and 8th respectively among all *Drosophila* genes for potential involvement in the JAK-STAT pathway (Supplemental Table S2). Neither gene scored positively in the JAK-STAT screen. However, the *upd3* gene is required in the fat-body-specific activation of the *Drosophila* JAK-STAT pathway [46], and the *BRWD3* gene is a positive regulator of JAK-STAT pathway found in a third-party RNAi screen [47]. Hence, the prioritized gene-function pairs we provided could serve as a useful resource to identify pathway components that might otherwise be missed by RNAi screening alone.

To examine the quality of our predictions in greater detail on a specific example, we compared prediction results for the *Drosophila* c-Jun N-terminal kinase (JNK) pathway to a recently published RNAi dataset specific for JNK pathway activations [48]. We found four genes (*nec, CG7338, Rac2* and *Mnn1*) in the top 5% by prediction score that were shown to result in activation of JNK signaling when knocked down by RNAi (Supplemental Table S5). The confidence scores we assigned to the four genes roughly correlated with the strength of JNK activation signals upon RNAi of these genes. The *nec* and *CG7338* genes were among our top predictions for the JNK pathway (0.872 and 0.639, ranked 1st and 3rd respectively, Supplemental Table S2) and showed high JNK activation when knocked down by RNAi. *Rac2* had a lower confidence score (0.338) and lower JNK activation signal, while *Mnn1* had the lowest confidence score and JNK signal.

## Discussion

The goal of function prediction is to infer novel functions for genes to help prioritize hypothesis-driven experimentation. The available evidence allows current prediction methods to quantify the shades of gray in gene function annotation. Therefore, the goal of experiment-prioritization is better approached not by assigning functions to genes in a binary fashion, but rather by *prioritizing* the most promising novel gene-function associations for future small-scale investigations [19], [49], [50]. Here we have provided a genome-wide resource of prioritized associations between *Drosophila* genes and their potential biological functions. Our model also helps to indicate false negatives in RNAi screening results, which often arise due to limited sensitivity in RNAi experiments [45] caused by both limited RNAi efficiency and tissue specificity of gene expression. High-scoring predictions not already verified in published screens could become interesting candidates for higher-sensitivity validation experiments, as suggested in our comparison with systematic RNAi screening data.

For the experimentalist users of function predictions, the quality of the predictions is an important concern. Currently available models generally rely on cross-validation for quality assessment of model performance, although some models use literature-mining and small-scale forward genetics assays to characterize the quality of a few prioritized prediction results [51]. We demonstrated the high performance of our prediction model relative to independent large-scale RNAi results that were not used in training our computational models. The significant overlap between our computational predictions and RNAi screening results suggests that RNAi screen results can be improved by using computational predictions to guide experiments, performing RNAi screens in size-reduced, prioritized subspaces predicted by our model, thus allowing more tissue types or experimental conditions to be tested with the same resources.

## Materials and Methods

### Sources of datasets

Genome-wide gene expression data across different developmental stages of *Drosophila* was obtained from the Berkeley *Drosophila* Genome Project (BDGP) [7] (www.fruitfly.org), which provided the expression data collected during *Drosophila* embryogenesis, and from a dataset [28] which covered the life cycle of *Drosophila*. Additional expression data for Drosophila genes were obtained from a variety of published datasets[52]–[74] at NCBI Gene Expression Omnibus (GEO) (Supplemental Table S7). Genome-wide gene sequences were downloaded from NCBI RefSeq database [32]. GO terms and gene-term associations were downloaded from the February 2009 snapshot of Gene Ontology database [38] (www.geneontology.org). Genetic interactions were obtained from BioGRID (www.thebiogrid.org) and FlyBase (www.flybase.org) [29], [30]. Physical protein-protein interactions in *Drosophila* were obtained from the Comprehensive Drosophila Interactions Database v5.0 (DroID, www.droidb.org) [27]. Conserved protein domain information was downloaded from InterPro (www.ebi.ac.uk/interpro/) [31]. Pathway annotations were downloaded from Kyoto Encyclopedia of Genes and Genomes (KEGG, www.genome.jp/kegg) [39].

### Random Forest algorithm, prediction model and prior features

We used the randomForest package for R-language (http://cran.r-project.org/web/packages/randomForest/index.html) [75] to train and test our prediction model. For each functional category (either a KEGG pathway or GO term) a Random Forest classifier was trained, and for each classifier 500 decision trees were generated. We left other parameters at default levels as defined by the randomForest package.

For the protein-protein interaction feature, pair-wise shortest path on the protein-protein interaction network was calculated using the Johnson Algorithm [76] as provided by the Boost Graph Library (www.boost.org/libs/graph/, http://search.cpan.org/perldoc?Boost::Graph). For microarray expression data, we calculated Pearson-Correlation Coefficients (average method) to measure the correlation between the expression profiles [7], [28] of a pair of genes. For genetic interactions we used binary values (0 or 1) to describe the existence of a genetic interaction (no matter whether it was a phenotypic enhancement or suppression) between a pair of genes. We also used the shortest path between two genes on the genetic interaction network as an additional feature of genetic interactions.

The *genetic interaction profile* for a given gene **X** was a binary vector describing the existence of genetic interactions between **X** and all other genes. The genetic interaction profile feature for a pair of genes was the Pearson-Correlation Coefficient (average method) between their genetic interaction profiles. Genetic interaction profiles could help to identify genes in the same pathway whose function was also achieved by another redundant pathway (Supplemental Figure S1A).

The *shared protein domains* feature includes two values: 1) the number of conserved protein domains shared between two proteins **X** and **Y**, denoted as **S**; and 2) the ratio between S and the total number (**U**) of unique domains on the two proteins **X** or **Y**. The *protein domain profile* for a given gene **X** was a binary vector describing the existence of each human protein domain in the IntePro database on gene **X**. The protein domain profile feature for a pair of genes was the Pearson-Correlation Coefficient (average method) between their protein domain profiles.

Sequence similarity was calculated using BLASTp [33] based on the Reciprocal Best Hits (RBH) approach [77]. The sequence similarity feature included two scores: pair-wise similarity and cross-species functional inference. To calculate the pair-wise similarity score, we counted the number (**S**) of shared orthologs between two genes **X** and **Y**, as well as the size (**U**) of the union of all their orthologs. The sequence similarity score for **X** and **Y** was then calculated as **S/U**. To calculate the score for cross-species function inference, for each candidate gene **X** and a given function **F**, we counted the number (**A**) of orthologs of gene **X** known to be associated with function **F**, and the number (**B**) of all its orthologs, and the score was then calculated as **A/B**. To prevent circularity caused by learning the functions of gene **X** from the functions of its orthologs in the same KO (KEGG Orthology) group to which **X** belonged, sequence similarity was not calculated between genes within the same KO group in KEGG. Similarly, ISS-tagged (Inferred from Sequence Similarity) gene-term associations in Gene Ontology were also excluded from the training and test sets.

### Model training and performance evaluation

The combination space of genes and functions was randomly split into 10 subsamples, with one designated as the test set, the others as training sets. For each function K_i_ a classifier C_i_ was trained and used in the test set to predict candidate genes for function K_i_. The process was repeated ten times so that each subsample was used as the test set once. All the predictions were then compared to known associations to calculate the performance. To make novel predictions, the model was trained in the entire known space of gene-function pairs and run in the entire unknown space of gene-function pairs.

### Keyword-matching between function prediction and RNAi screening hits

From the titles of the 24 published DRSC RNAi screens, we chose keywords which can be found in the descriptions of corresponding KEGG pathways or GO terms (Supplemental Table S6). For those instances where one RNAi screen was matched to multiple KEGG pathways or GO terms, we considered these pathways or terms as one general function matched to the screen. For any gene **X** found as a positive hit in a given RNAi screen, all the positive predictions associating gene **X** with the matched KEGG pathways or GO terms were counted as successful recoveries of the hit.

### Canonical supervised learning model built for comparison purpose

To compare the performance of our model in recovering positive hits in RNAi screens with the canonical supervised learning model, we trained a Random Forest model with the same prior features and training sets used in our model. In this model, objects were gene-function pairs, labels were either “yes” or “no” (indicating whether a gene-function pair was labeled as a valid association or not), and features for a given gene-function pair were a variety of measures (see Materials and Methods) of similarity between the given gene and all positive reference genes for that function. All gene-function pairs in the training set were used to train a single classifier which was used to make predictions in the test set of gene-function pairs. These predictions were then compared to the results of RNAi screens. The performance of the canonical model in recovering positive RNAi hits was compared to that of the function-specific model (Figure 5).

### Code performance and comparison with canonical supervised machine-learning model

We executed our code on a Dell PowerEdge server equipped with 32 GBytes of RAM and 8 Intel Xeon 2.8 GHz processors running Red Hat Linux Advanced Server 64 bit Edition 4. The training of our function-specific classifier model took approximately 43 minutes for KEGG pathway membership prediction and 3 hours 57 minutes for GO term prediction (compared to the performance of the canonical model which required 3 hours 16 minutes for KEGG pathway prediction and 15 hours 20 minutes for GO term prediction, Supplemental Figure S2).

## Supporting Information

Table S1 Classification of KEGG pathways.
(0.04 MB XLS)

Table S2 GO term and KEGG pathway membership predictions with confidence scores above 0.2 (for GO term predictions) or above 0.1 (for KEGG pathway predictions).
(3.91 MB XLS)

Table S3 KEGG pathway predictions matched to DRSC screening.
(0.03 MB XLS)

Table S4 GO-term predictions matched to DRSC screening.
(0.04 MB XLS)

Table S5 JNK pathway prediction compared to RNAi data at the Japan National Institute of Genetics (NIG).
(0.03 MB DOC)

Table S6 Keyword matching between RNAi screens and gene functions.
(0.02 MB XLS)

Table S7 Additional Expression Profiles from NCBI Gene Expression Omnibus (GEO).
(0.10 MB XLS)

Figure S1 Genetic interaction profiles. A, Both pathway P1 and P2 drive downstream processes to achieve a function; loss of genes within either pathway will not abolish the function. However, when a pair of genes from the two pathways respectively are lost (e.g. a-x, a-y, c-x), both pathways will be broken and a loss-of-function phenotype will emerge. B, Genes within the same pathway have similar genetic interaction profiles, which could be useful in categorizing a,b,c and x,y,z into pathway P1 and P2 respectively.
(1.07 MB TIF)

Figure S2 Code performance of Functional-Specific Classifier model and canonical supervised machine-learning model.
(0.51 MB TIF)

Figure S3 Performance of GO prediction when removing one feature at a time from the model. Receiver Operating Characteristic curves (A) and Precision-Recall curves (B) for the overall performance and the performance when removing one feature at a time in GO term (biological process, BP) prediction. Precision-Recall curves for the GO term prediction model for GO terms with various degrees of specificity, i.e., those that have been annotated with 0–25 genes (C), 25–50 genes (D), 50–100 genes (E), and 100–500 genes (F).
(1.73 MB TIF)

Figure S4 Performance of KEGG prediction when removing one feature at a time from the model. Receiver Operating Characteristic curves (A) and Precision-Recall curves (B) for the overall performance and the performance when removing one feature at a time in the KEGG pathway prediction. Precision-Recall curves for the performance of the model in predicting metabolism only (C), signaling pathway only (D), basic functions (E), and all non-metabolism functions (F).
(2.20 MB TIF)

Figure S5 Performance of KEGG prediction when limited in the gene space of genetic interaction network. Receiver Operating Characteristic curves (A) and Precision-Recall curves (B) for the overall performance and contribution of each feature in the KEGG pathway prediction. Precision-Recall curves for the performance of the model in predicting metabolism only (C), signaling pathway only (D), basic functions (E), and all non-metabolism functions (F).
(1.85 MB TIF)
