# Supplementary material for: A Genome-Wide Gene Function Prediction Resource for Drosophila melanogaster
Source: PLoS One. 2010 Aug 12;5(8):e12139. doi: 10.1371/journal.pone.0012139 (PMC2920829; doi:10.1371/journal.pone.0012139)
Supplement: Table S5 — JNK pathway prediction compared to RNAi data at the Japan National Institute of Genetics (NIG). (0.03 MB DOC) [file pone.0012139.s005.doc]

| ***Flybase ID*** | ***CG*** | ***symbol*** | ***score*** | *JNK activation strength and location (Umemori et al, 2009)* | | | | |
| --- | --- | --- | --- | --- | --- | --- | --- | --- |
|  |  |  |  | **N** | **DB** | **DC** | **DM** | **VM** |
| FBgn0002930 | CG1857 | nec | 0.872 | +- | ++ | ++ | ++ | + |
| FBgn0037073 | CG7338 | CG7338 | 0.639 | ++ | ++ | ++ | ++ | ++ |
| FBgn0014011 | CG8556 | Rac2 | 0.338 |  | + | + | + | +- |
| FBgn0031885 | CG13778 | Mnn1 | 0.146 |  | +- | +- | +- | +- |

Supplemental Table S5 | **JNK pathway activations in recent RNAi screening matched with our prediction results.** N: Notum, DB: Dorsal wing Blade, DC: DorsoCentral spot, DM: Dorsal wing Margin, VM: Ventral wing Margin, VB: Ventral wing Blade(Umemo*ri et* al, 2009).
