# Supplementary figures and images for: A Genome-Wide Gene Function Prediction Resource for Drosophila melanogaster
Source: PLoS One. 2010 Aug 12;5(8):e12139. doi: 10.1371/journal.pone.0012139 (PMC2920829; doi:10.1371/journal.pone.0012139)

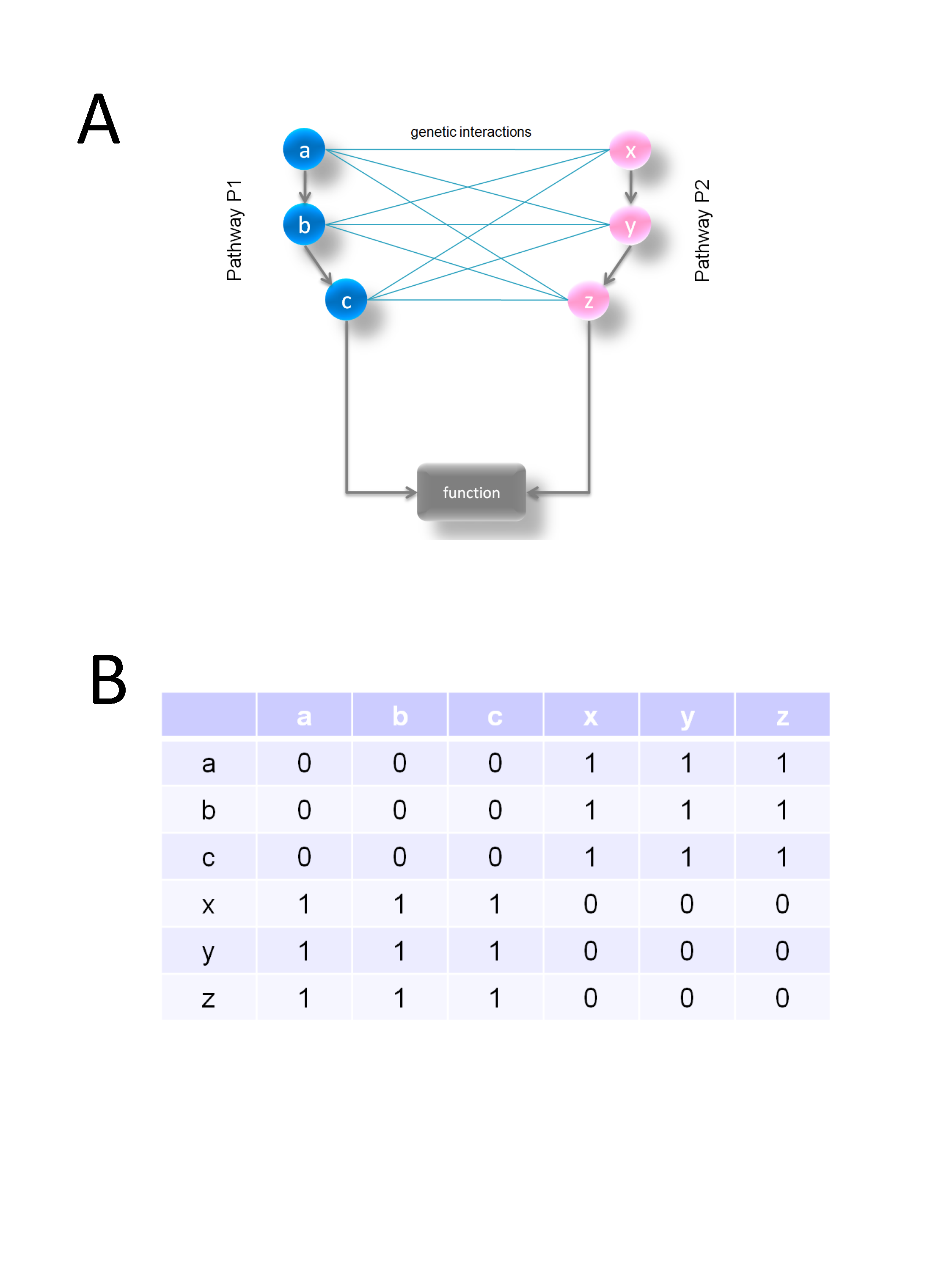

Supplement: Figure S1 — Genetic interaction profiles. A, Both pathway P1 and P2 drive downstream processes to achieve a function; loss of genes within either pathway will not abolish the function. However, when a pair of genes from the two pathways respectively are lost (e.g. a-x, a-y, c-x), both pathways will be broken and a loss-of-function phenotype will emerge. B, Genes within the same pathway have similar genetic interaction profiles, which could be useful in categorizing a,b,c and x,y,z into pathway P1 and P2 respectively. (1.07 MB TIF) [file pone.0012139.s008.tif]

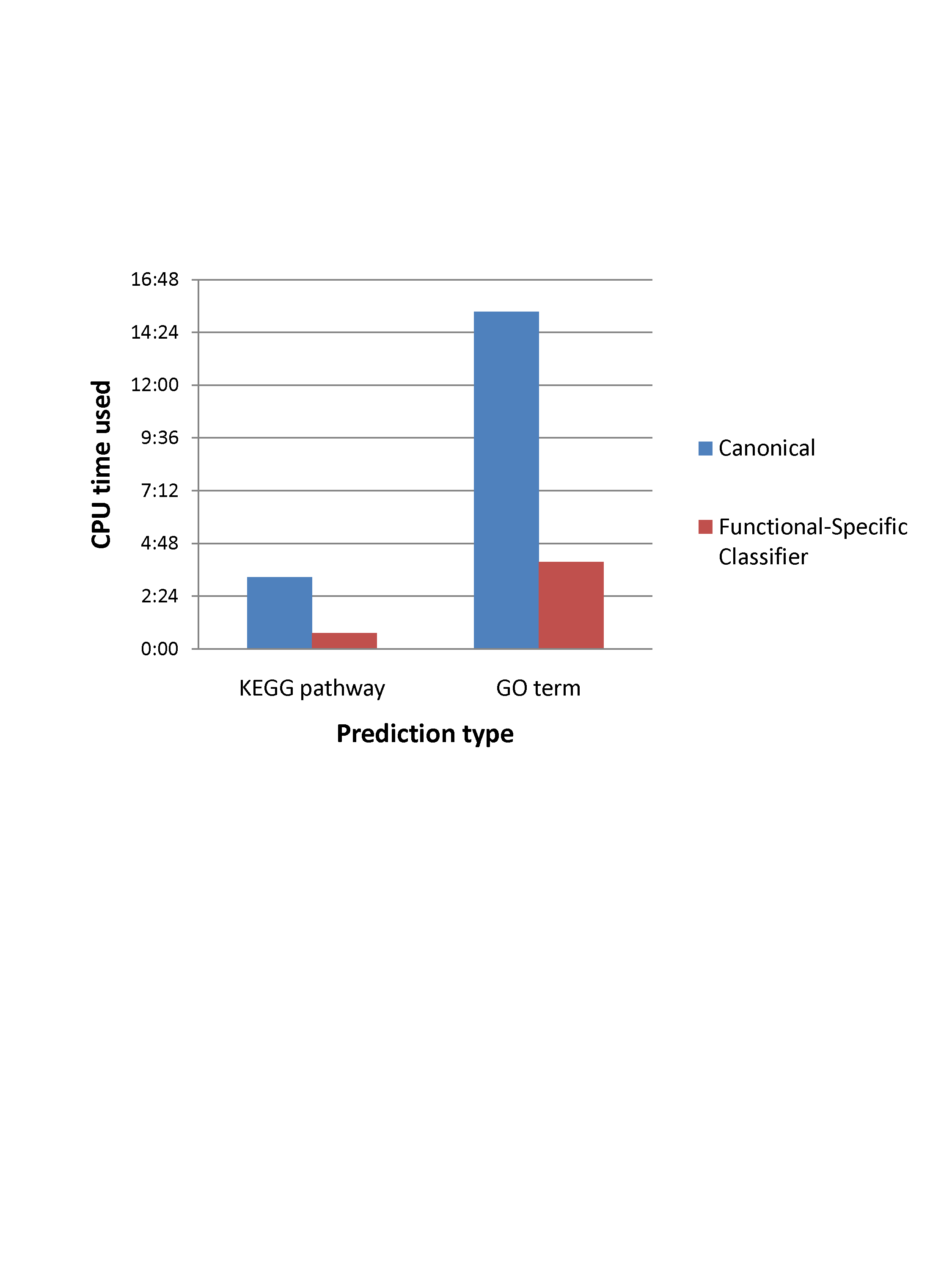

Supplement: Figure S2 — Code performance of Functional-Specific Classifier model and canonical supervised machine-learning model. (0.51 MB TIF) [file pone.0012139.s009.tif]

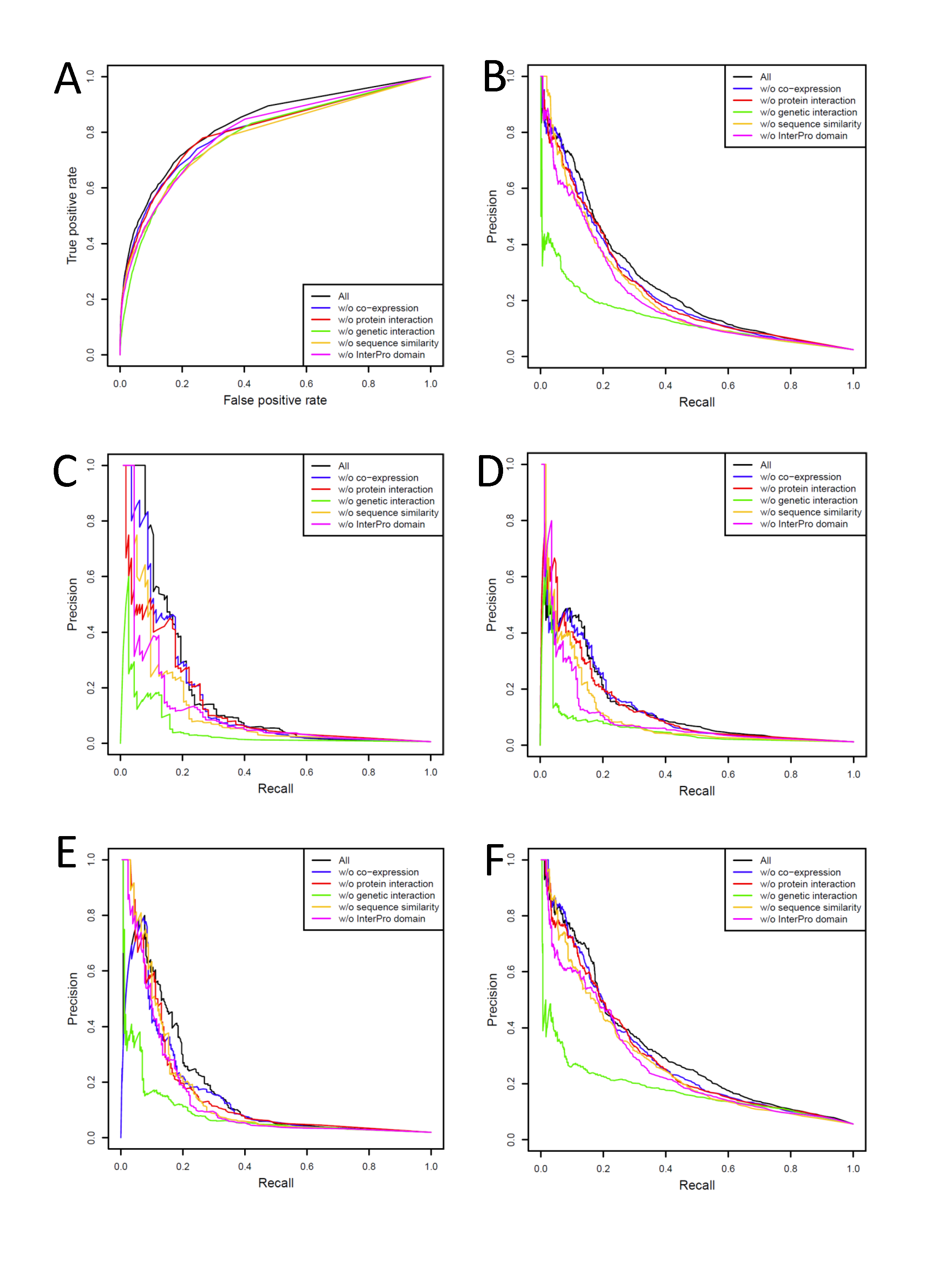

Supplement: Figure S3 — Performance of GO prediction when removing one feature at a time from the model. Receiver Operating Characteristic curves (A) and Precision-Recall curves (B) for the overall performance and the performance when removing one feature at a time in GO term (biological process, BP) prediction. Precision-Recall curves for the GO term prediction model for GO terms with various degrees of specificity, i.e., those that have been annotated with 0–25 genes (C), 25–50 genes (D), 50–100 genes (E), and 100–500 genes (F). (1.73 MB TIF) [file pone.0012139.s010.tif]

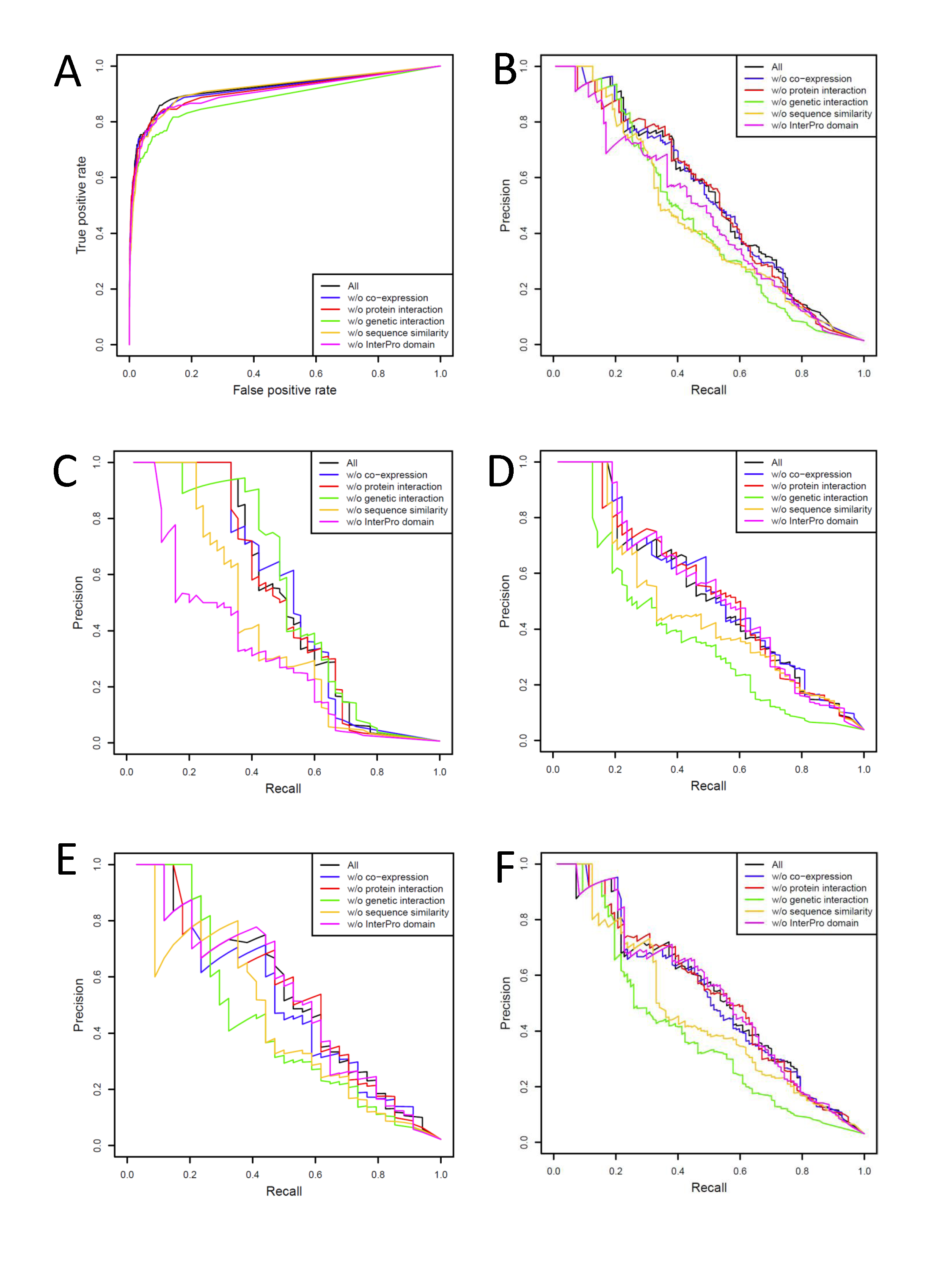

Supplement: Figure S4 — Performance of KEGG prediction when removing one feature at a time from the model. Receiver Operating Characteristic curves (A) and Precision-Recall curves (B) for the overall performance and the performance when removing one feature at a time in the KEGG pathway prediction. Precision-Recall curves for the performance of the model in predicting metabolism only (C), signaling pathway only (D), basic functions (E), and all non-metabolism functions (F). (2.20 MB TIF) [file pone.0012139.s011.tif]

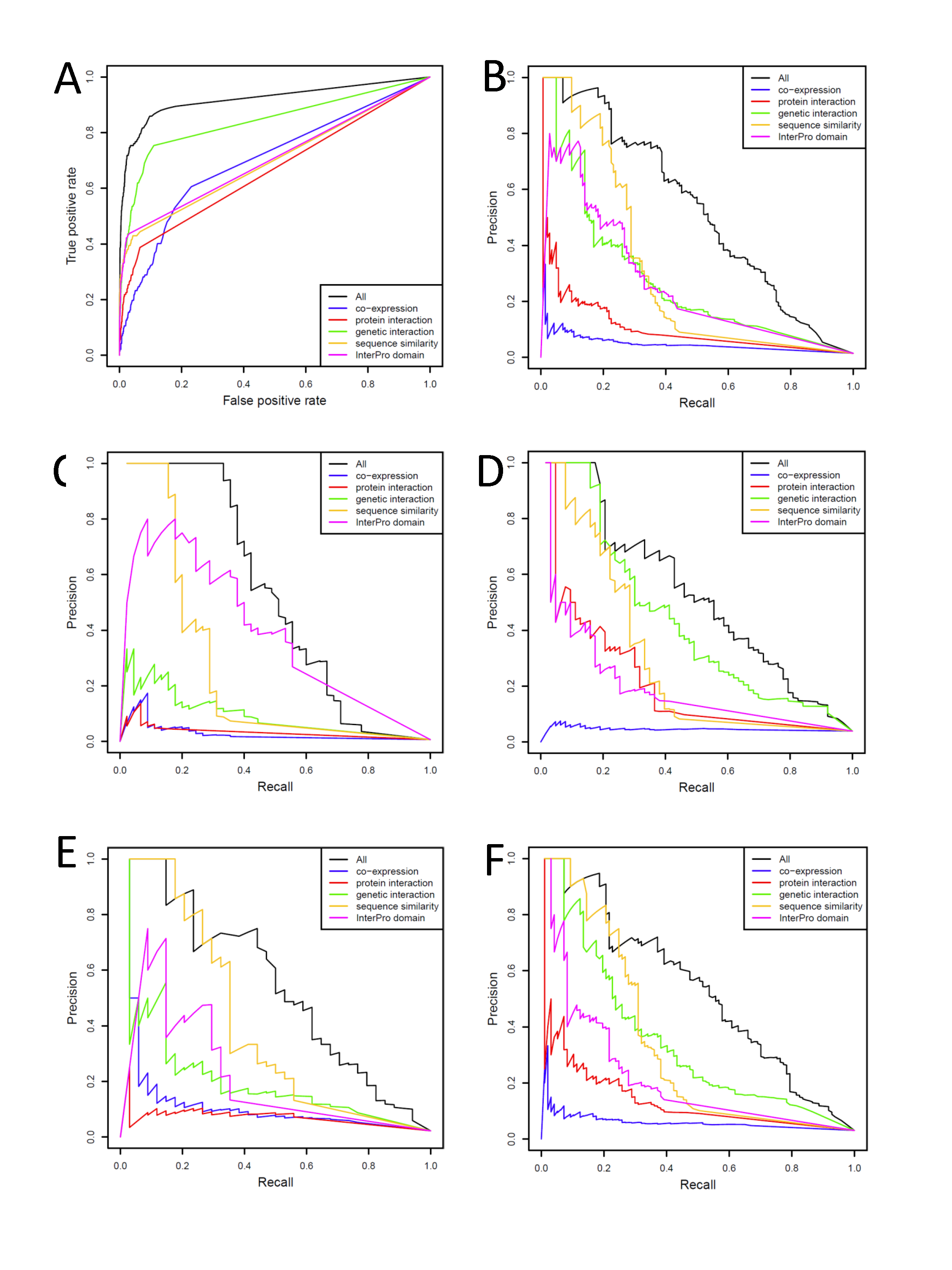

Supplement: Figure S5 — Performance of KEGG prediction when limited in the gene space of genetic interaction network. Receiver Operating Characteristic curves (A) and Precision-Recall curves (B) for the overall performance and contribution of each feature in the KEGG pathway prediction. Precision-Recall curves for the performance of the model in predicting metabolism only (C), signaling pathway only (D), basic functions (E), and all non-metabolism functions (F). (1.85 MB TIF) [file pone.0012139.s012.tif]
